# NKL homeobox gene MSX1 acts like a tumor suppressor in NK-cell leukemia

**DOI:** 10.18632/oncotarget.18609

**Published:** 2017-06-21

**Authors:** Stefan Nagel, Claudia Pommerenke, Corinna Meyer, Maren Kaufmann, Roderick A.F. MacLeod, Hans G. Drexler

**Affiliations:** ^1^ Department of Human and Animal Cell Lines, Leibniz-Institute DSMZ, Braunschweig, Germany

**Keywords:** homeobox, NKL-code, T-ALL

## Abstract

NKL homeobox gene MSX1 is physiologically expressed in lymphoid progenitors and subsequently downregulated in developing T- and B-cells. In contrast, elevated expression levels of MSX1 persist in mature natural killer (NK)-cells, indicating a functional role in this compartment. While T-cell acute lymphoblastic leukemia (T-ALL) subsets exhibit aberrant overexpression of MSX1, we show here that in malignant NK-cells the level of MSX1 transcripts is aberrantly downregulated. Chromosomal deletions at 4p16 hosting the MSX1 locus have been described in NK-cell leukemia patients. However, NK-cell lines analyzed here showed normal MSX1 gene configurations, indicating that this aberration might be uncommon. To identify alternative MSX1 regulatory mechanisms we compared expression profiling data of primary normal NK-cells and malignant NK-cell lines. This procedure revealed several deregulated genes including overexpressed IRF4, MIR155HG and MIR17HG and downregulated AUTS2, EP300, GATA3 and HHEX. As shown recently, chromatin-modulator AUTS2 is overexpressed in T-ALL subsets where it mediates aberrant transcriptional activation of MSX1. Here, our data demonstrate that in malignant NK-cell lines AUTS2 performed MSX1 activation as well, but in accordance with downregulated MSX1 transcription therein we detected reduced AUTS2 expression, a small genomic deletion at 7q11 removing exons 3 and 4, and truncating mutations in exon 1. Moreover, genomic profiling and chromosomal analyses of NK-cell lines demonstrated amplification of IRF4 at 6p25 and deletion of PRDM1 at 6q21, highlighting their potential oncogenic impact. Functional analyses performed via knockdown or forced expression of these genes revealed regulatory network disturbances effecting downregulation of MSX1 which may underlie malignant development in NK-cells.

## INTRODUCTION

Human blood cells originate in the bone marrow where hematopoietic stem cells (HSC) generate ancestors of both the myeloid and lymphoid lineages. The common lymphoid progenitors (CLP) differentiate into B-cells, T-cells or natural killer (NK)-cells. Naïve B-cells terminate their maturation in lymph nodes, early T-cell progenitors migrate for subsequent differentiation into the thymus, while NK-cells usually complete their development in the bone marrow [1]. The developmental processes of lymphopoiesis are mainly regulated at the transcriptional level [2, 3]. Accordingly, lymphocyte differentiation depends on activities of particular transcription factors (TFs) like PAX5 for B-cells, BCL11B for T-cells, and ID2, NFIL3 and STAT5 for NK-cells [4, 5].

NK-cell lymphocytes together with additional innate lymphoid cells (ILCs) belong to the fast-acting innate immune system and protect against pathogens and cancer [6]. Malignancies derived from the NK-cell lineage are rare and have a dismal prognosis [7, 8]. Chromosomal and genomic analyses of primary malignant NK-cells have revealed several recurrent aberrations [9–11], indicating that these alterations contribute to the process of transformation in this cell type. In cancer cells the processes of proliferation, apoptosis and differentiation are frequently disturbed [12]. Accordingly, in NK-cell malignancies dysregulation of these processes has been imputed to aberrant expression of PRDM1, MYC, and IRF8, respectively [13–15]. Of note, NK-cell lines proved instrumental in the identification and analysis of genomic alterations and deregulated genes, supporting their usage for basic research of this cancer [16]. Nevertheless, the genesis of this tumor type is still far from clear.

Malignant cells of T-cell acute lymphoblastic leuke-mia (T-ALL) are developmentally arrested thymocytes expressing stage-specific genes and particular oncogenes [17]. Homeobox genes TLX1, TLX3, NKX2-1 and NKX2-5 encode oncogenic TFs in T-ALL which are physiologically silenced during hematopoiesis, but undergo ectopic activation in transformed thymocytes [18]. They belong to the NKL subclass of homeobox genes which numbers to date 20 aberrantly expressed members in T-ALL [19]. Although deregulated NKL homeobox genes have been described in B-cell malignancies as well, this gene subclass plays its major oncogenic role in T-cell leukemia [19–21]. However, their exact role(s) in leukemogenesis is still unclear although impacts on proliferation, survival, genomic integrity and differentiation have been described [19, 22–26].

Homeobox genes regulate fundamental processes in both embryonal development and differentiation in the adult. Some represent master genes for specific cell types/organs like NKX2-3 (spleen), NKX2-5 (heart), or NKX3-1 (prostate), others operate a code which regulates the development of complex structures or tissues [27–30]. Accordingly, we coined the term NKL-code which describes the physiological expression pattern of NKL homeobox genes in early hematopoiesis and lymphopoiesis [19]. Due to their basic impact, aberrant activity patterns of NKL homeobox genes potentially contribute to leukemogenesis/lymphomagenesis by deregulating developmental processes.

MSX1 belongs to the NKL subclass, is physiologically expressed in CLPs, downregulated in the course of T-cell development and aberrantly activated in T-ALL [19, 31]. In this T-cell malignancy MSX1 is variously (de)regulated via the BMP-pathway and AUTS2. BMP-signalling inhibits the expression of MSX1 but aberrant repression of this pathway impels MSX1 activation in T-ALL subsets [32]. AUTS2 is a modifier of polycomb repressor complex 1 (PRC1) and operates by interaction with component PCGF5 and recruitment of histone acetyltransferase EP300 [33, 34]. Aberrant overexpression of AUTS2 activates homeobox gene MSX1 by converting repressor variant PRC1.5 into an activator [34, 35].

In this study we analyzed the expression and regulation of NKL homeobox gene MSX1 in NK-cell leukemia. Our data indicate that MSX1 serves as a tumor suppressor (TS) gene in NK-cells contrasting the situation in T-cells. Using NK-cell lines as models we identified deregulated upstream factors forming a regulatory network and mobilizing developmental perturbations in this lymphoid malignancy.

## RESULTS

### Downregulation of NKL homeobox gene MSX1 in NK-cell leukemia

**Figure 1:**
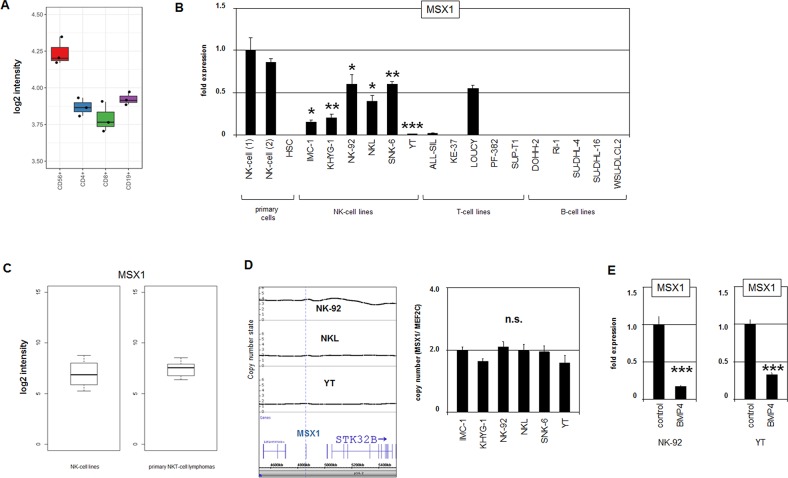
MSX1 in NK-cells **(A)** Expression profiling data for MSX1 of CD56+ NK-cells, CD4+ T-cells, CD8+ T-cells and CD19+ B-cells obtained from dataset GSE72642 are depicted in a boxplot. The expression level in NK-cells is significantly higher when compared to the other lymphocytes (p=0.040, Mann-Whitney U Test). **(B)** RQ-PCR analysis of MSX1 expression in samples of primary NK-cells of two different donors and of HSCs, and in 6 NK-cell lines, 5 T-cell lines and 5 B-cell lines. Data demonstrate aberrantly reduced MSX1 levels in malignant NK-cells. The values and statistics are given in relation to primary NK-cells. **(C)** Expression data for MSX1 of 4 NK-cell lines and 19 primary NK/T-cell lymphoma patient samples (GSE19067). The data show no significant statistical difference, indicating similar expression levels. **(D)** Copy number analysis of the MSX1 locus in NK-cell lines NK-92, NKL and YT by genomic profiling (left). Please note, NK-92 possesses a hypo-tetraploid karyotype, therefore, elevated copy numbers at 4p16 reflects the whole genome of this cell line. Copy number analysis of the MSX1 locus was performed in comparison to the control-locus of MEF2C at 5q14 in NK-cell lines by RQ-PCR (right). **(E)** Quantification of MSX1 transcription by RQ-PCR after treatment of NK-cell lines NK-92 and YT with BMP4, demonstrating an inhibitory impact of BMP-signalling on MSX1 expression.

Recently, we showed that MSX1 is active in CLPs and that T- and B-lymphocytes downregulate this homeobox gene during their development while NK-cells maintain increased transcriptional activity [19]. Here, examination of a public profiling dataset of primary normal peripheral lymphocytes (GSE72642) confirmed elevated MSX1 transcript levels in CD56+ NK-cells and reduced levels in CD4+ T-cells, CD8+ T-cells and CD19+ B-cells (Figure 1A). However, quantification of MSX1 expression in a panel of six malignant NK-cell lines (IMC-1, KHYG-1, NK-92, NKL, SNK-6, YT) by RQ-PCR revealed reduced levels when compared to primary normal NK-cells obtained from two normal donors (Figure 1B). Furthermore, expression levels of MSX1 quantified in selected lymphoid cell lines showed (physiologically) downregulated gene activity in T- and B-cell lines. In contrast, the T-cell line LOUCY demonstrated aberrantly enhanced activation. These data indicate aberrant inhibition of MSX1 in NK-cell leukemia, while MSX1 overexpression was detectable in T-ALL subsets as shown previously [32]. Moreover, the expression levels of MSX1 in NK-cell lines were similar when compared to primary NK/T-cell lymphoma patient samples (Figure 1C), supporting our observation of aberrantly reduced MSX1 activity in NK-cell malignancies. Accordingly, MSX1 represents an oncogene in T-cell leukemia and presumably a TS gene in NK-cell leukemia.

Patient samples from NK-cell malignancies showed several genomic abnormalities including deletion of the chromosomal region 4p16 hosting MSX1 [11, 13]. However, copy number analysis of MSX1 by genomic profiling in three NK-cell lines, namely NK-92, NKL and YT, and quantitative PCR analysis of the panel of six NK-cell lines demonstrated normal gene configurations (Figure 1D, Supplementary Figure 1A). Thus, genomic aberrations are not generally responsible for the observed suppression of MSX1 gene activity, indicating the presence of additional deregulated upstream factors in malignant NK-cells.

### Comparative expression profiling revealed potential regulators of MSX1

To identify upstream factors involved in deregulated MSX1 gene activity in NK-cell malignancies we compared gene expression profiling datasets from three different donors of primary normal CD56+ NK-cells (GSE72642) with those from five malignant NK-cell lines: IMC-1, KHYG-1, NK-92, SNK-6 and YT (GSE19067 and GSE53478). Gene-annotation enrichment analysis of the top 1000 differentially expressed genes highlighted upregulation of the cell cycle and downregulation of NK-cell cytotoxicity in malignant NK-cells, connoting proliferation and differentiation, two basic processes generally disturbed in cancer. Furthermore, the data indicated activation of diverse metabolic pathways and inhibition of several signaling pathways (Supplementary Figure 2). Of note, this analysis revealed no hint of aberrant activity of the BMP-pathway which has been shown to inhibit MSX1 in the T-cell lineage [32]. Nevertheless, treatment of NK-cell lines NK-92 and YT with BMP4 resulted in reduced MSX1 expression levels, replicating the regulatory impact described in T-cells (Figure 1E).

This comparative profiling approach indicated in leukemic NK-cells 2040 upregulated and 3403 downregulated genes Supplementary Tables 1A, 1B, respectively showing at least 4-fold differentially expressed transcript levels. Not all of these genes are related to malignancy. Nevertheless, these quantities suggest that suppression represents an important type of gene deregulation in this type of cancer. Furthermore, screening of both gene lists revealed conspicuous candidates which plausibly might be involved in MSX1 deregulation including upregulated IRF4, MIR155HG, MIR17HG and SMAD3, and downregulated AUTS2, EP300, GATA3, HHEX, IL7R and JARID2.

### Aberrant expression of AUTS2 contributes to MSX1 deregulation

**Figure 2:**
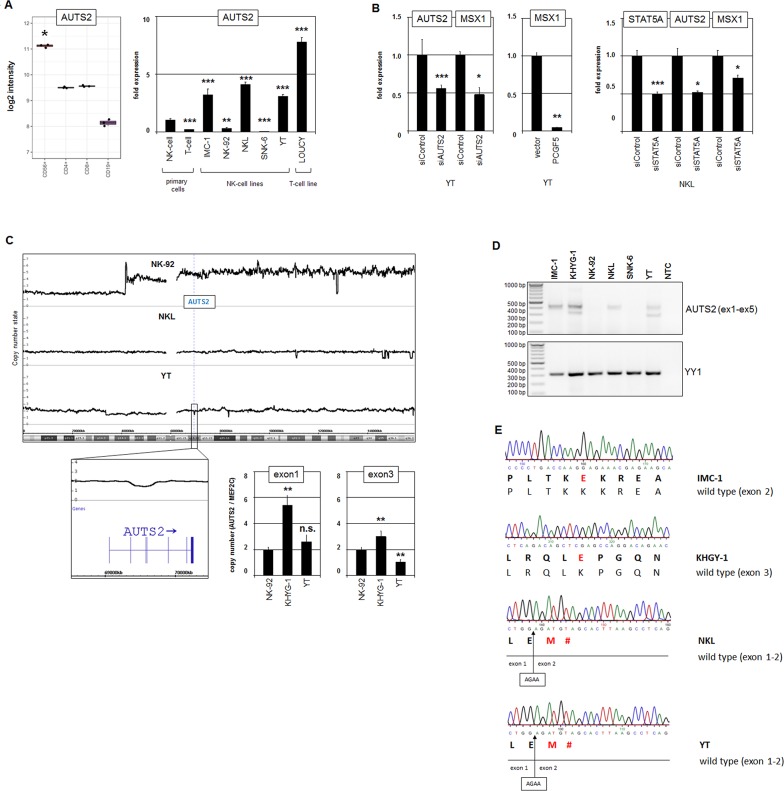
Analysis of AUTS2 in NK-cell lines **(A)** Expression profiling data for AUTS2 of CD56+ NK-cells, CD4+ T-cells, CD8+ T-cells and CD19+ B-cells obtained from dataset GSE72642 are depicted as a boxplot (left). RQ-PCR analysis of AUTS2 expression in primary NK-cells and T-cells and 5 NK-cell lines and T-cell line LOUCY which has been shown to express aberrantly enhanced AUTS2 (right). The values and statistics are given in relation to primary NK-cells. **(B)** RQ-PCR analysis of MSX1 expression after siRNA-mediated knockdown of AUTS2 in YT cells (left), after forced expression of PCGF5 (middle), and RQ-PCR analysis of AUTS2 and MSX1 expression after siRNA-mediated knockdown of STAT5A in NKL cells (right). **(C)** Copy number analysis of the AUTS2 locus in NK-cell lines by genomic profiling (above) and RQ-PCR (below). Data indicate a small deletion targeting exons 3 and 4 of AUTS2 in YT cells. RQ-PCR data indicate genomic deletion of exon 3 in KHYG-1 cells as well. **(D)** RT-PCR analysis of AUTS2 exons 1 to 5 (above) and of YY1 for control (below). Note an additional smaller PCR product in KHYG-1 and YT cells. **(E)** Sequence analysis of RT-PCR products revealed particular AUTS2 mutations in the NK-cell lines IMC-1, KHYG-1, NKL and YT.

The discovery of deregulated AUTS2 attracted our interest because this gene encodes a chromatin-modulator which activates MSX1 expression in T-cells and might thus play a similar role in normal/malignant NK-cells as well [35]. Figure 2A depicts expression data of AUTS2 for primary NK-, T- and B-cells, indicating a prominent role in normal NK-lymphocytes. We analyzed AUTS2 in more detail and quantified its transcripts in malignant NK-cell lines in comparison to primary normal NK-cells (Figure 2A). However, while two cell lines showed reduced levels (NK-92, SNK-6), three expressed even raised AUTS2 levels (IMC-1, NKL, YT) indicating aberrant variability.

Furthermore, siRNA-mediated knockdown of AUTS2 in NK-cell line YT resulted in concomitantly reduced expression of MSX1 (Figure 2B), demonstrating that AUTS2 activates MSX1 in NK-cells as described in T-cells. Forced expression of PCGF5 (the repressive partner of AUTS2) resulted in strongly reduced MSX1 expression (Figure 2B), supporting the regulatory mode of action of AUTS2 via PRC1.5 [33, 34]. Moreover, siRNA-mediated knockdown of STAT5A resulted in suppression of AUTS2 in addition to MSX1 (Figure 2B), showing that STAT5 activates AUTS2 expression in both NK- and T-cells [35].

Genomic profiling of the AUTS2 gene in three NK-cell lines indicated absence of amplification or deletion of its locus at 7q11, giving no explanation for varying expression levels (Figure 2C). However, in YT cells we detected a small deletion targeting just exons 3 and 4 in one allele which was confirmed by RQ-PCR analysis (Figure 2C). Qualitative RT-PCR analysis comprising the exons 1 to 5 of 6 NK-cell lines confirmed low/absent AUTS2 expression levels in NK-92 and SNK-6 cells and showed an additional smaller band in KHYG-1 and YT cells. RQ-PCR analysis of KHYG-1 showed marked copy number gain for exon1 which was less pronounced for exon 3 (Figure 2C), indicating AUTS2 gene duplication and concomitant exon 3 deletion. Thus, these data show absence of exons 3 and/or 4 in a fraction of the AUTS2 transcripts from these two cell lines (Figure 2D).

To look for mutations in this conspicuous region of the AUTS2 gene we sequenced the RT-PCR-products from the four high level expressing NK-cell lines as shown in Figure 2D. This analysis revealed in each cell line wild type configurations in addition to particular mutations (Figure 2E). IMC-1 contained a point-mutation in exon 2 resulting in amino acid exchange K124E. A similar mutation, K176E, was detected in exon 3 in KHYG-1 cells. In NKL we detected a short deletion of 4 base pairs which resulted in a frame-shift generating a stop-codon immediately downstream. In YT we confirmed the absence of exons 3 and 4 in the small transcript type. Moreover, this transcript contained this short deletion as detected in NKL (Figure 2E). Taken together, low expression levels and particular gene mutations of AUTS2 may curtail its activating function and thus contribute to reduced transcription of its target gene MSX1 in malignant NK-cells.

### Combination of expression and genomic profiling data

**Figure 3:**
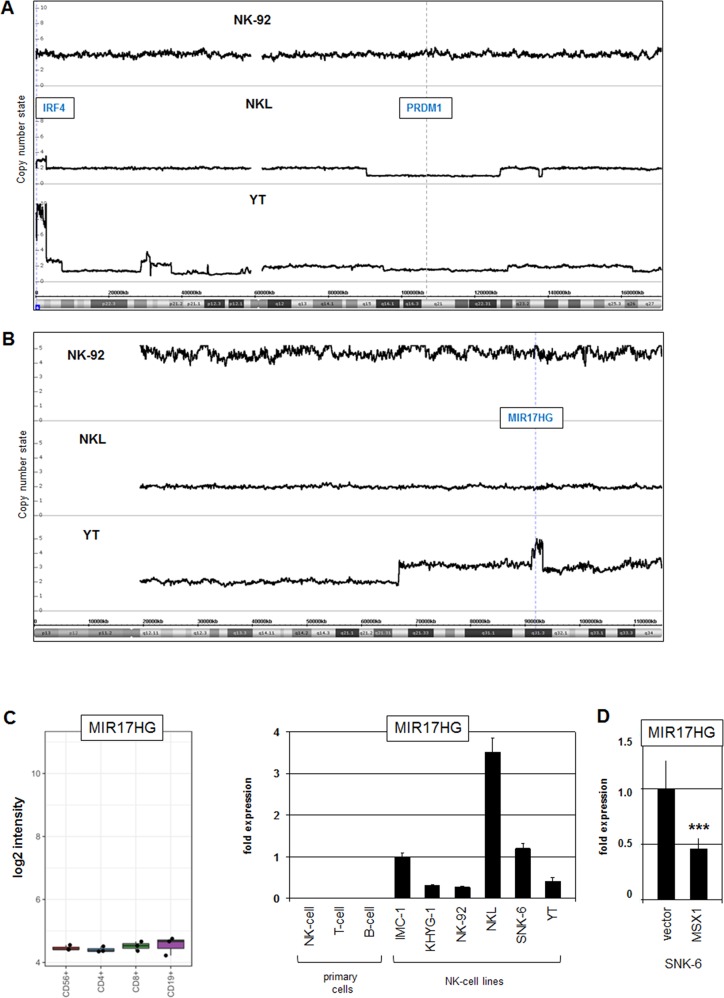
Genomic aberrations at chromosomes 6 and 13 in NK-cell lines **(A)** Copy number analysis by genomic profiling of NK-cell lines NK-92, NKL and YT revealed aberrations at chromosome 6 in NKL and YT. Conspicuous loci are highlighted, including amplification of IRF4 at 6p25 and deletion of PRDM1 at 6q21. Of note, NK-92 is a hypo-tetraploid cell line. **(B)** Genomic profiling of NK-cell lines NK-92, NKL and YT revealed an amplicon in YT containing the locus for MIR17HG at 13q31. **(C)** Expression profiling data for MIR17HG of CD56+ NK-cells, CD4+ T-cells, CD8+ T-cells and CD19+ B-cells obtained from dataset GSE72642 are depicted in a boxplot (left). RQ-PCR analysis of MIR17HG expression in primary NK-, T- and B-cells and 6 NK-cell lines (right). **(D)** Forced expression of MSX1 in SNK-6 cells resulted in reduced expression of MIR17HG.

Next, we analyzed our genomic profiling data from the three NK-cell lines, NK-92, NKL and YT, to see if copy number changes correlated with abnormal gene activities. This procedure revealed subterminal amplification of chromosome 6 at position 6p25 containing overexpressed IRF4 in NKL and YT cells (Figure 3A). In addition, the same chromosome showed long-arm deletions in NKL and YT, targeting the known TS gene PRDM1 at 6q21 (Figure 3A). Furthermore, two amplicons were detected in YT cells, one located at 13q31 containing MIR17HG (Figure 3B), and the other at 15q22 containing SMAD3 (Supplementary Figure 1B, 1C).

MIR17HG encodes a cluster of six oncogenic micro-RNAs playing a role in T-ALL and is regulated by NKL homeodomain proteins [23, 36]. Examination of expression profiling data from normal primary peripheral lymphocytes (GSE72642) demonstrated equally low levels of MIR17HG in NK-, T- and B-cells (Figure 3C). In contrast, RQ-PCR analysis of MIR17HG showed enhanced transcript levels in all six NK-cell lines as compared to primary normal NK- and T-cells which tested negative (Figure 3C), confirming aberrant activation in malignant NK-cells. To see if the described activating impact of NKL homeodomain proteins operates in NK-cells as well, we overexpressed MSX1 in SNK-6 cells. This procedure resulted in decreased expression levels of MIR17HG, showing that MSX1 inhibits this miR-gene cluster in NK-cells (Figure 3D). Thus, amplification of MIR17HG and reduced levels of its repressor MSX1 contribute to elevated expression of this oncogene.

SMAD3 encodes a TF and represents a component of the TGFbeta-pathway. However, in the malignant context SMAD3 is activated by BMP-signalling [37] which inhibits MSX1 expression in NK-cells as shown above (Figure 1D). Thus, its overexpression might enhance this repressive effect of the BMP-pathway. In contrast, reduced expression levels of the IL7-receptor (IL7R) might result in decreased STAT5 activity which is located downstream of IL7R. Of note, the IL7-STAT5 pathway is important for NK-cell development and activates the MSX1-activator AUTS2 (Figure 2B) [4, 35]. Thus, reduced expression of IL7R might perturb both MSX1 transcription and the differentiation process in NK-cells. However, our genomic profiling data do not indicate a loss of gene copies for IL7R at 5p13 (Supplementary Figure 1D), excluding chromosomal aberrations as potential explanation for its reduced expression level.

Next, we focussed on the known NK-cell differen-tiation factor PRDM1 and on IRF4 which has not been described in this context so far. Furthermore, we analyzed the remaining shortlisted gene candidates MIR155HG, JARID2, EP300, GATA3, and HHEX in more detail.

### Tumor suppressor PRDM1 activates the expression of MSX1

**Figure 4:**
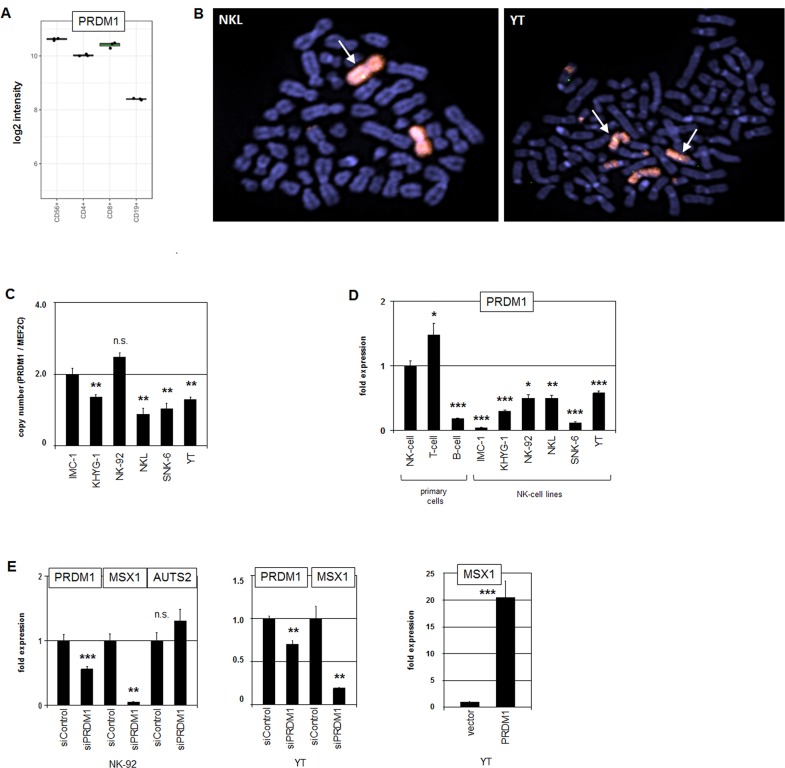
Analysis of PRDM1: expression, genomics and target gene regulation **(A)** Expression profiling data for PRDM1 of CD56+ NK-cells, CD4+ T-cells, CD8+ T-cells and CD19+ B-cells obtained from dataset GSE72642 are depicted in a boxplot. **(B)** FISH analysis of the PRDM1 locus in NKL (left) and YT cells (right). The PRDM1 probe was labelled green (appearing yellow) and the probe for the whole chromosome 6 red. PRDM1 loci are indicated by an arrow. Both cell lines exhibit a loss of one PRDM1 allele at 6q21. **(C)** Copy number analysis of the PRDM1 locus in NK-cell lines by RQ-PCR indicates a loss of PRDM1 in KHYG-1, NKL, SNK-6 and YT but not in IMC-1 and NK-92. The values and statistics are given in relation to IMC-1. **(D)** RQ-PCR analysis of PRDM1 expression in primary NK- and T-cells and six NK-cell lines revealed suppressed transcript levels in malignant NK-cells. The values and statistics are given in relation to primary NK-cells. **(E)** RQ-PCR analysis of PRDM1, MSX1 and AUTS2 expression after siRNA-mediated knockdown of PRDM1 in NK-92 cells (left), and RQ-PCR analysis of PRDM1 and MSX1 expression after siRNA-mediated knockdown of PRDM1 in YT cells (middle). RQ-PCR analysis of MSX1 expression after forced expression of PRDM1 in YT cells (left). The data indicate that PRDM1 activates MSX1 transcription.

PRDM1/BLIMP1 encodes a TF that is physio-logically expressed in NK-cells, T-cells and B-cells (Figure 4A). In NK-cells PRDM1 mediates their maturation and represents a TS gene in NK-cell malignancies [38]. FISH analysis confirmed deletion of PRDM1 loci in the cell lines NKL and YT (Figure 4B). Genomic RQ-PCR analysis of the PRDM1 gene in six NK-cell lines indicated decreased copy numbers in KHYG-1, NKL, SNK-6 and YT (Figure 4C). Accordingly, RQ-PCR analysis of PRDM1 transcripts in all NK-cell lines demonstrated reduced expression levels when compared to primary NK- and T-cells (Figure 4D). However, reduced PRDM1 expression in IMC-1 and NK-92 was taken to indicate additional suppressive mechanisms (see below).

To examine the potential impact of PRDM1 on MSX1 we performed siRNA-mediated knockdown of PRDM1 in NK-92 and YT cells. Concomitant downregulation of MSX1 demonstrated that PRDM1 is an activator of MSX1 (Figure 4E). Knockdown of PRDM1 did not alter the expression level of AUTS2, indicating that PRDM1 does not regulate MSX1 transcription via this chromatin-modulator. Furthermore, forced expression of PRDM1 in YT cells resulted in enhanced transcription of MSX1 (Figure 4E), endorsing an activating role for PRDM1. Thus, genomic deletions at 6q21 correlated with low PRDM1 expression levels in malignant NK-cell lines contributing to reduced transcription of MSX1.

### Overexpressed IRF4 inhibits MSX1 and activates MIR155HG

**Figure 5:**
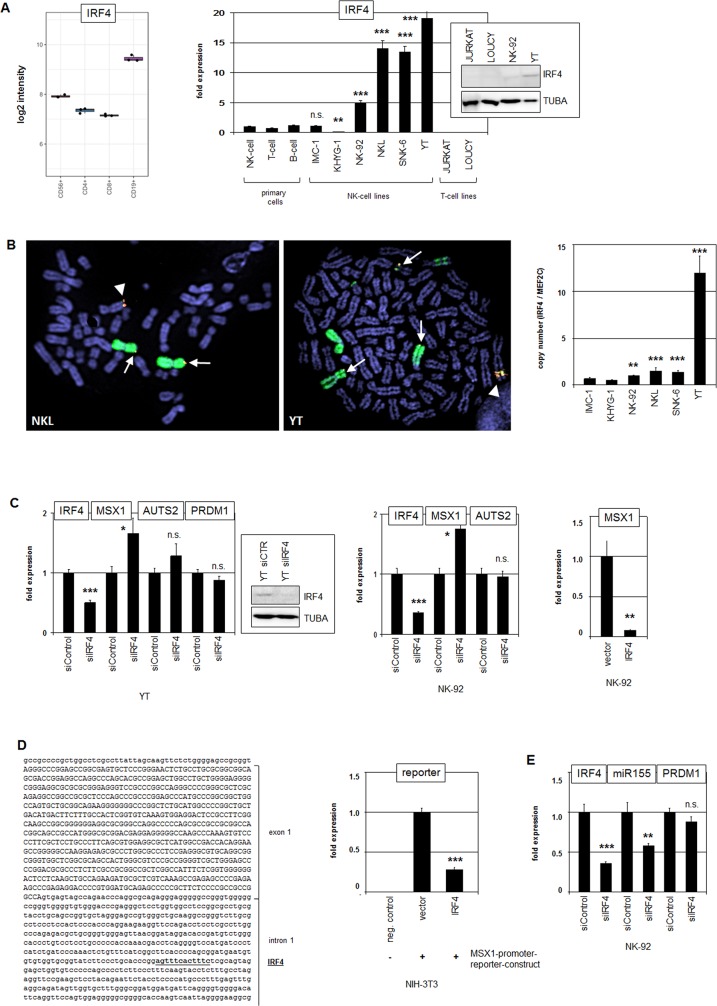
Analysis of IRF4: expression, genomics and target gene regulation **(A)** Expression profiling data for IRF4 of CD56+ NK-cells, CD4+ T-cells, CD8+ T-cells and CD19+ B-cells obtained from dataset GSE72642 are depicted in a boxplot (left). RQ-PCR analysis of IRF4 expression in primary NK- and T-cells and 6 NK-cell lines revealed enhanced transcript levels in NK-cell lines NK-92, NKL, SNK-6 and YT (right). The values and statistics are given in relation to primary NK-cells. Western blot analysis demonstrates elevated IRF4 expression at the protein level in NK-92 and YT cells (insert), correlating with transcription data. T-cell lines JURKAT and LOUCY served as controls. **(B)** FISH analysis in NKL (left) and YT (middle), demonstrating amplification and translocation of the IFR4 locus. The IRF4 probe was labelled red and the probe for the whole chromosome 6 was labelled green. Arrows indicate normal IRF4 loci and arrowheads translocated/amplified IRF4 loci. Copy number analysis of the IRF4 locus in NK-cell lines was performed by RQ-PCR (right). **(C)** RQ-PCR analysis of IRF4, MSX1, AUTS2 and PRDM1 expression after siRNA-mediated knockdown of IRF4 in YT cells (left) and in NK-92 cells (middle). Knockdown of IRF4 expression was additionally shown at the protein level by Western blot in YT cells (box). Forced expression of IRF4 in NK-92 cells resulted in repression of MSX1 as analyzed by RQ-PCR (right). **(D)** Reporter gene assay of the intronic MSX1 region (left) indicates direct regulation of MSX1 by IRF4 demonstrating the repressive activity in NIH-3T3 cells (right). **(E)** RQ-PCR analysis of IRF4, MIR155HG, and PRDM1 expression after siRNA-mediated knockdown of IRF4 in NK-92 cells.

IRF4 encodes a TF of the IRF-family members of which regulate several immunological processes in lymphocytes [39]. IRF1, IRF2 and IRF8 are functionally important in NK-cells while IRF4 plays a prominent role in B-cells as supported by elevated expression levels therein (Figure 5A) [39, 40]. Quantification of IRF4 transcript levels by RQ-PCR in malignant NK-cell lines in comparison to primary normal NK-cells demonstrated enhanced expression in NK-92, NKL, SNK-6 and YT while IMC-1 showed equal and KHYG-1 reduced levels (Figure 5A). Western blot analysis showed higher IRF4 protein amounts in YT than in NK-92 while control T-cell lines JURKAT and LOUCY tested negative (Figure 5A), confirming the elevated expression in NK-cell lines at the protein level. Chromosomal analysis of the IRF4 locus at 6p25 by FISH confirmed a copy number gain in NKL and amplification in YT (Figure 5B), as indicated by genomic profiling. In addition, the IRF4-gain/amplicon was aberrantly translocated in both cell lines demonstrating a similar and more complex mode of rearrangement. Copy number quantification of IRF4 by PCR in six NK-cell lines indicated circa 6 copies in YT and duplications in NK-92, NKL and SNK-6 as compared to IMC-1 (Figure 5B). These genomic data correlated with enhanced expression levels in the cell lines. Thus, copy number gain represents an important mechanism for aberrantly increased IRF4 expression in malignant NK-cells.

To analyze if IRF4 is also involved in MSX1 regulation we performed siRNA-mediated knockdown of IRF4 in YT and NK-92 cells (Figure 5C). Subsequent RQ-PCR analysis demonstrated elevated MSX1 transcript levels, indicating that IRF4 mediates suppression of MSX1. Concomitant quantification of AUTS2 and PRDM1 transcripts showed no significant alterations, showing that MSX1 regulation by IRF4 is not mediated via these factors. However, sequence analysis of the MSX1 gene revealed a potential binding site for IRF4 in the non-coding region of intron 1 [41]. To check if IRF4 interacts with this identified site we performed a reporter gene assay for a corresponding fragment of the MSX1 intron in NIH-3T3 cells (Figure 5D). These data showed reduced reporter gene activity in the presence of IRF4, demonstrating an inhibitory and direct impact of IRF4 on MSX1 via this site. Thus, IRF4 is overexpressed in malignant NK-cells and operates as a suppressor of MSX1 transcription.

MIR155HG encodes the micro-RNA miR155, represents a known target gene of IRF4 [42], and was consistently identified as an overexpressed gene candidate in our profiling approach (Supplementary Table 1A). Accordingly, siRNA-mediated knockdown of IRF4 in NK-92 cells resulted in decreased expression levels of MIR155HG, confirming that IRF4 serves to activate MIR155HG in malignant NK-cells (Figure 5E). However, PRDM1 was not regulated by IRF4 in NK-cell lines (Figure 5E), contrasting with the situation in T-cells [43].

### Analysis of MIR155HG, EP300, GATA3 and HHEX

**Figure 6:**
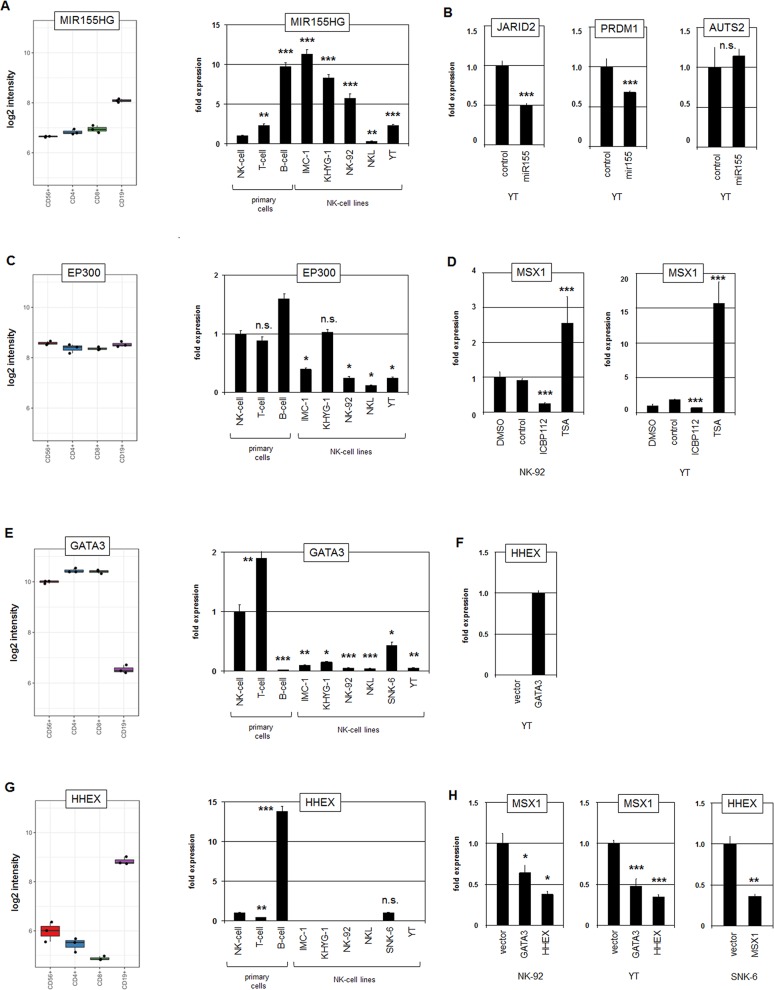
Analysis of MIR155HG, EP300, GATA3 and HHEX **(A)** Expression profiling data for MIR155HG of CD56+ NK-cells, CD4+ T-cells, CD8+ T-cells and CD19+ B-cells obtained from dataset GSE72642 are depicted in a boxplot (left). RQ-PCR analysis of MIR155HG expression in primary NK- and T-cells and five NK-cell lines (right). The values and statistics are given in relation to primary NK-cells. **(B)** RQ-PCR analysis of JARID2, PRDM1 and AUTS2 expression in YT cells after transfection with a microRNA-mimic for miR155 shows an inhibitory impact of miR155 on JARID2 and PRDM1 but not on AUTS2. **(C)** Expression profiling data for EP300 of CD56+ NK-cells, CD4+ T-cells, CD8+ T-cells and CD19+ B-cells obtained from dataset GSE72642 are depicted in a boxplot (left). RQ-PCR analysis of EP300 expression in primary NK- and T-cells and 5 NK-cell lines (right). The values and statistics are given in relation to primary NK-cells. **(D)** RQ-PCR analysis of MSX1 after treatment of NK-92 cells (left) and YT cells (right) with histone acetyltransferase inhibitor ICBP112 and histone deacetylase inhibitor TSA. The values and statistics are given in relation to the DMSO-control. **(E)** Expression profiling data for GATA3 of CD56+ NK-cells, CD4+ T-cells, CD8+ T-cells and CD19+ B-cells obtained from dataset GSE72642 are depicted in a boxplot (left). RQ-PCR analysis of GATA3 expression in primary NK- and T-cells and six NK-cell lines (right). The values and statistics are given in relation to primary NK-cells. **(F)** RQ-PCR analysis of HHEX after forced expression of GATA3 in NK-92 cells showing induction of HHEX expression. **(G)** Expression profiling data for HHEX of CD56+ NK-cells, CD4+ T-cells, CD8+ T-cells and CD19+ B-cells obtained from dataset GSE72642 are depicted in a boxplot (left). RQ-PCR analysis of HHEX expression in primary NK- and T-cells and six NK-cell lines (right). The values and statistics are given in relation to primary NK-cells. **(H)** RQ-PCR analysis of MSX1 after forced expression of GATA3 and HHEX in NK-92 and YT cells (left and middle), and of HHEX after forced expression of MSX1 in SNK-6 cells (right).

Profiling data from normal primary peripheral lymphocytes (GSE72642) revealed the lowest expression level of MIR155HG in NK-cells (Figure 6A). In contrast, RQ-PCR analysis of MIR155HG showed enhanced transcript levels in five NK-cell lines as compared to primary normal NK-cells (Figure 6A), confirming aberrant activation therein. Genomic profiling data for NK-92, NKL and YT showed normal gene configurations at 21q21, indicating that copy number gain plays no prominent role in aberrant activation of MIR155HG (Supplementary Figure 1E). Of note, SNK-6 expressed extremely high levels of MIR155HG (data not shown), suggesting the operation of more regulators in this particular cell line, possibly including EBV-mediated activation [44]. To analyze potential targets of miR155 we transfected an RNA-mimic for miR155 into YT cells. This procedure was accompanied by decreased transcript levels of the known miR155-target JARID2 [45], as well as of PRDM1 while AUTS2 RNA levels showed no significant alterations (Figure 6B). Of note, decreased JARID2 expression levels were detected by our comparative profiling approach (Supplementary Table 1B), supporting its downregulation by overexpressed MIR155HG. Consistently, the online tool TargetScan identified a miR155-3p binding site in the 3’-UTRs of JARID2 as well as in PRDM1 but not for AUTS2 (data not shown). Finally, the enhanced expression levels of MIR155HG inversely correlated with PRDM1 transcript levels in B-cells and NK-cell lines. Thus, miR155 probably inhibits the expression of MSX1-activator PRDM1.

EP300 encodes a histone acetyltransferase which interacts with AUTS2 to transform the repressor PRC1.5 into an activator [34]. Expression profiling data of normal peripheral lymphocytes showed no differences for EP300 transcript levels (Figure 6C), discounting cell-type specific gene activity in lymphocytes. In contrast, RQ-PCR analysis confirmed decreased expression in NK-cell lines (Figure 6C), showing aberrant downregulation. However, genomic profiling data for NK-92, NKL and YT showed normal gene configurations for EP300 at 22q13 (Supplementary Figure 1F), excluding underlying genomic aberrations. Treatment of NK-cell lines NK-92 and YT with inhibitors of histone acetyltransferase (ICBP112) and histone deacetylase (TSA) resulted in reduced and enhanced MSX1 expression, respectively (Figure 6D), confirming an activating role for acetylated histones on MSX1 transcription. Thus, downregulation of EP300 contributes to a reduction of MSX1 expression.

Finally, our comparative profiling analysis revealed reduced expression levels of GATA3 and HHEX in malignant NK-cells as compared to normal NK-cells (Supplementary Table 1B). Recently, we have shown in T-cells that GATA3 mediates activation of HHEX which in turn activates MSX1 [19]. To analyze this relationship in NK-cells we quantified their expression levels in primary normal lymphocytes and NK-cell lines (Figure 6E, 6G), thus confirming aberrantly decreased transcript levels of GATA3 and HHEX in malignant NK-cell lines. However, genomic profiling data of NK-92, NKL and YT showed normal gene configurations for GATA3 and HHEX at 10p14 and 10q23, respectively, indicating that copy number alterations do not play a role in their aberrant suppression in these cell lines (Supplementary Figure 1G). Furthermore, we overexpressed GATA3 and HHEX in NK-cell lines. Subsequent RQ-PCR analysis showed induction of HHEX expression in YT cells confirming HHEX activation by GATA3 (Figure 6F). But surprisingly, the results indicated inhibition of MSX1 expression by GATA3 and HHEX (Figure 6H). These findings contrast with the situation observed in T-cells [19]. Moreover, forced expression of MSX1 in SNK-6 cells resulted in decreased HHEX transcription (Figure 6H), demonstrating mutual repression of HHEX and MSX1 in NK-cells. Thus, in both T- and NK-cells the TFs encoding genes GATA3, HHEX and MSX1 constitute a regulatory network which, however, differ in a cell-type specific manner with respect to MSX1. Taken together, analyses of identified gene candidates revealed that overexpression of MIR155HG (PRDM1 suppressor) and downregulation of EP300 (AUTS2 cofactor) contribute to reduced MSX1 transcript levels. GATA3 and HHEX represent downregulated MSX1 repressors which are therefore not involved in aberrant MSX1 suppression in NK-cells.

## DISCUSSION

**Figure 7:**
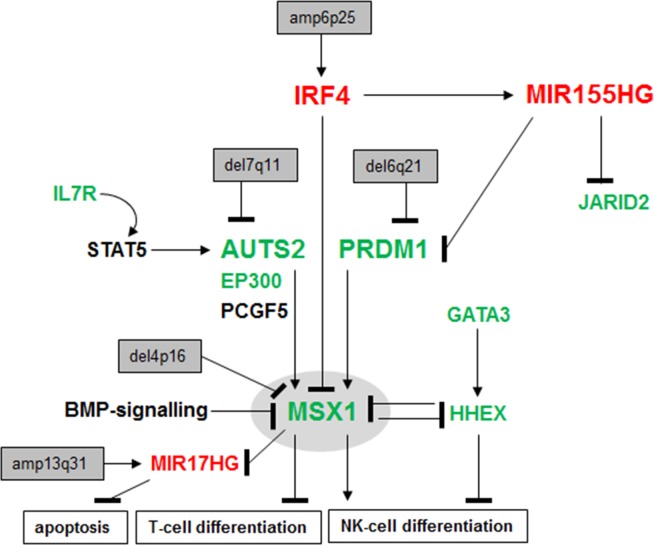
Deregulated gene regulatory network in NK-cell leukemia This figure summarizes the results obtained in this study. NKL homeobox gene MSX1 represents a TS in NK-cell malignancies, mediates NK-cell differentiation and inhibits T-cell differentiation as described previously [35]. Deletions at 4p16 which might target the MSX1 locus have been described in NK-cell leukemia patients [11, 13]. Aberrantly expressed upstream factors involved in MSX1 deregulation include IL7R, AUTS2, IRF4, MIR155HG and PRDM1. Deregulated MIR17HG has been described to suppress apoptosis in T-cells [23]. Overexpressed genes are indicated in red, downregulated genes in green.

The results of this study are summarized in Figure 7, showing a gene regulatory network which mediates aberrant differentiation in malignant NK-cells. Here, we analyzed the expression of NKL homeobox gene MSX1 in NK-cell lines and identified deregulated upstream factors. MSX1 is physiologically expressed in early stages of lymphopoiesis and during NK-cell development [19, 31]. The respective aberrant up- and down-regulation of MSX1 in T-ALL [32, 35] and NK-cell malignancies indicate contrasting oncogene and TS roles in these related lymphoid entities. In NK-cell malignancies several chromosomal alterations have been identified including genomic deletion at 4p16 [11, 13]. While this aberration might target MSX1 at this position, NK-cell lines investigated here showed normal genomic configurations of the MSX1 gene, alerting to alternative means of dysregulation.

AUTS2 is a chromatin-modulator and activator of homeobox gene MSX1 in T-cells and NK-cells as described here [35]. AUTS2 interacts with PRC1-component PCGF5 and turns this repressor complex into an activator by recruitment of histone acetyltransferase EP300 [33, 34]. Overexpression of AUTS2 in T-ALL mediates aberrant activation of MSX1 in subsets of this malignancy [35]. We found reduced expression levels of AUTS2 in two NK-cell lines which might result in concomitantly reduced MSX1 transcript levels therein. In AUTS2 overexpressing NK-cell lines we found several gene abnormalities including deletion of exons 3 and 4, mutations predicted to result in truncated AUTS2 protein, and non-synonymous gene mutations changing charged amino acid residues. Of note, according to an alignment of vertebrate AUTS2 protein sequences, the identified AUTS2 mutations alter conserved amino acids, supporting their functional importance (Supplementary Figure 3). Moreover, genomic deletions of 5’-exons have been described in patients with neurodevelopmental disorders, and an aberrant gene fusion of 5’-PAX5 and 3’-AUTS2 in B-ALL patients results in losses of anterior parts of AUTS2 protein [46–49]. These findings highlight the functional importance of the N-terminal AUTS2 protein as indicated here in NK-cells. In contrast, isoform analysis and scoring of disease severities highlight C-terminal AUTS2 domains in neurodevelopmental contexts [50]. Nevertheless, high AUTS2 expression levels in normal NK-cells, reduced expression levels and the presence of several AUTS2 gene abnormalities in malignant NK-cells indicates that this developmental regulator plays an important role in lymphopoiesis and may activate particular differentiation genes in addition to MSX1.

STAT5 activated AUTS2 expression in malignant NK- and T-cells and represents an important factor in normal NK-cell development [35, 51, 52]. Interestingly, activating mutations have been detected in the IL7-receptor gene IL7R, the mediating kinase JAK3, and its target protein STAT5B in T-cell leukemia [35, 53, 54]. These data support the idea of aberrantly activated IL7-STAT5-AUTS2-MSX1 signalling in leukemic T-cells and suggest aberrant suppression of this pathway in malignant NK-cells. However, activating mutations of STAT5B have been described in some cases of NK/T-cell lymphoma as well [55], generating opposing interpretations. In NK-cell lines activating mutations were detected in STAT3 but not in STAT5B genes [56]. Therefore, the search for potential loss-of-function mutations in the IL7R and STAT5 encoding genes in NK-cell malignancies represents an ongoing research project in this laboratory. Of note, DDX3X encodes an RNA-helicase and contains frequently inactivating mutations in NK/T-cell lymphoma patients [55]. In addition to its described role in cell cycle control and inhibition of signalling pathways DDX3X may be involved in the regulation of NK-cell differentiation as well.

EP300 interacts with AUTS2 and performs gene activation via histone acetylation. This interaction occurs via C-terminal regions of AUTS2 [34]. Accordingly, truncated AUTS2 proteins as reported here are unable to interact with EP300. Histone acetylation regulates MSX1 gene activity as described here in NK-cells, and as shown previously in the context of T-cells and neural crest cells [35, 57], endorsing this type of chromatin modification as a general requirement for MSX1 transcription. Histone deacetylases (HDAC) counteract the function of histone acetyltransferases. Accordingly, HDAC inhibitor TSA activated the expression of MSX1. Significantly, while the HDAC-inhibitor induced apoptosis in malignant NK-cells an inhibitor of histone acetyltransferases showed no such effect (Supplementary Figure 4). Therefore, treatment with HDAC inhibitors might represent a promising option for the therapy of NK-cell malignancies as shown for other cancer types [58].

Our data demonstrate that PRDM1 activates the transcription of MSX1 and is downregulated in NK-cells. Interestingly, PRDM1 is a regulator of NK-cell maturation, acts as a TS in NK-cell malignancies and is accordingly suppressed by genomic deletions, DNA-methylation, and particular mutations [11, 13, 59]. Moreover, PRDM1 is involved in the development of other lymphoid cell types including differentiation of NK/T-cell and B-cells [60, 61]. Consistently, PRDM1 acts as a TS gene in the ABC-type of diffuse large B-cell lymphoma, connoting poor prognosis if downregulated [62]. We propose that PRDM1 may act as an activator of MSX1 in early B-cell development as well because of simultaneous MSX1 gene activity in B-cell progenitors [19].

IRF1, IRF2, and IRF8 are regulators of NK-cell maturation [63, 64]. Accordingly, mutations in IRF8 impair the process of NK-cell differentiation [15]. In contrast, IRF4 is rather a regulator of early B-cells and of late T-cells [39, 65]. Both, IRF4 and IRF8 are involved in plasma cell differentiation and create sequential double-negative feedback loops to regulate B-cell differentiation [66, 67]. Here, we identified an amplicon at chromosomal position 6p25 which mediates overexpression of IRF4 in NK-cell lines. This chromosomal aberration has been described in NK-cell leukemia patients as well, supporting its clinical significance [11, 13]. Our data show that IRF4 is a direct repressor of MSX1 transcription highlighting this pathway as a mediator of aberrant deregulation. Furthermore, IRF4 represents an activator of MIR155HG in NK-cells as described previously in B-cells [42], supporting the general significance of this regulatory interaction. Although IRF4 activates the expression of PRDM1 in T-cells [43], this was not confirmed by our NK-cell line experiments, but is consistent with data obtained from primary NK-cells [38]. Finally, IRF4 has been described as an oncogene in HTLV1-positive T-cell leukemia and in multiple myeloma [64]. Accordingly, our data support an oncogenic function for IRF4 in NK-cells, thereby extending the transforming capacity of this gene in hematological cancers.

MIR155HG encodes the micro-RNA miR155 which is an oncogene in B-cell lymphomas and a target of EBV [68, 69]. SNK-6 is an EBV-positive NK-cell line which consistently expresses high levels of MIR155HG as noted here [44]. Our data indicate that PRDM1 is a novel target of miR155 in NK-cells which might be the case in other cell types as well. Furthermore, MSX1 inhibits the micro-RNA cluster gene MIR17HG in NK-cells contrasting with the situation in T-cells where NKL homeodomain proteins activate MIR17HG expression [23]. Thus, genomic amplification of MIR17HG and reduction of its transcriptional repressor MSX1 results in activation of MIR17HG expression in malignant NK-cells. These data suggest that MIR17HG performs oncogenic functions in both NK-cells and in T-cells [36].

GATA3 regulates HHEX and HHEX in turn regulates MSX1 in T-cells [19]. We observed here that these factors are mutual regulators in NK-cells as well. However, while the HHEX-MSX1 connection is stimulatory in T-cells it is suppressive in NK-cells. Of note, the impact of GATA-factors on HHEX represents a conserved regulatory relationship as shown recently in mice [70]. This gene regulatory network might be involved in the differentiation process of NK-cells and ILCs because GATA3 represents an important regulator at an early stage of development where these cell types differentiate [3]. HHEX and GATA3 have been shown to interact with each other, supporting their coordinated activity [71]. Furthermore, HHEX and MSX1 are members of the NKL subclass of homeobox genes and belong to the NKL-code in hematopoiesis, highlighting their role in lymphoid development [19]. However, the observed aberrant downregulation of these MSX1 repressors does not contribute to aberrantly decreased MSX1 levels in malignant NK-cell lines.

In conclusion, our results illuminate novel molecular mechanisms mediating transformation of NK- cell progenitors. The analyzed TS and oncogenes play physiological roles in the differentiation of B-, T- and NK-cell lymphocytes. Aberrant activities of involved networks and factors like suppression of MSX1 in lymphoid progenitors might represent an important step in the development of NK-cell malignancies.

## MATERIALS AND METHODS

### Cell lines and treatments

NK-cell lines KHYG-1, NK-92, NKL and YT have been reviewed by Drexler & Matsuo [16]. Together with T-cell lines JURKAT and LOUCY they are held by the DSMZ (Braunschweig, Germany). The NK-cell line IMC-1 was kindly obtained from Dr. I. Ming Chen (Albuquerque, NM, USA) and NK-cell line SNK-6 from Dr. N. Shimizu (Tokyo, Japan) [44, 72]. These NK-cell lines tested all CD3-negative and CD56-positive. Cell lines were cultivated as described elsewhere [73]. Cell stimulations were performed by treatment with recombinant human BMP4 for 16 h at a concentration of 20 ng/ml (R&D Systems, Wiesbaden, Germany), 10 μg/ml Trichostatin A (TSA) (Sigma, Taufkirchen, Germany), and 0.5 μM ICBP112 (Sigma). Gene specific siRNA oligonucleotides, AllStars negative Control siRNA (siControl) and miRNA-Mimics were obtained from Qiagen (Hilden, Germany). Expression constructs for GATA3, HHEX, IRF4, MSX1, PCGF5 and PRDM1 were cloned in vector pCMV6 and obtained from Origene (Wiesbaden, Germany). SiRNAs (80 pmol), miRNA-Mimic for miR155 (80 pmol), and expression constructs/vector controls (2 μg) were transfected into 1×10^6^ cells by electroporation using the EPI-2500 impulse generator (Fischer, Heidelberg, Germany) at 350 V for 10 ms. Transfected cells were harvested after 20 h cultivation.

### Genomic and chromosomal analyses

For genomic profiling of the NK-cell lines NK-92, NKL and YT genomic DNA was prepared by the Qiagen Gentra Puregene Kit (Qiagen). Labelling, hybridization and scanning were performed at the Genome Analytics Facility, Helmholtz Centre for Infection Research (Braunschweig, Germany), according to the manufacturer’s protocols (Affymetrix, High Wycombe, UK). Data were interpreted using the Chromosome Analysis Suite software version 2.0.1.2 (Affymetrix, High Wycombe, UK).

Chromosomal analysis by fluorescent in-situ hybridization (FISH) was performed as described previously [74]. BAC and fosmid clones were obtained from BacPac Resources, Children’s Hospital Oakland Research Institute (CA, USA) to analyze PRDM1 (G248P81739G4) and IRF4 (RP11-1072F10). Insert DNA was harvested using the Big BAC DNA Kit (Princeton Separations, Adelphia, NJ, USA) and directly labelled by nick translation with dUTP-fluors (Dyomics, Jena, Germany). Whole chromosome painting probes were obtained from Applied Spectral Imaging (Neckarhausen, Germany). Fluorescent images were captured and analyzed with an Axio-Imager microscope (Zeiss, Göttingen, Germany) configured to a dual Spectral Imaging FISH system (Applied Spectral Imaging).

### Expression profiling and bioinformatics

Public expression profiling datasets were obtained from Gene Expression Omnibus (GEO; www.ncbi.nlm.nih.gov/gds): GSE72642 for primary CD19-positive B-cells, CD4-positive T-cells, CD8-positive T-cells and CD56-positive NK-cells using the RosetteSep negative selection method for three different donors [75], GSE19067 for NK-cell lines IMC-1, KHYG-1, NK-92 and SNK-6 and for 19 primary NK/T-cell lymphoma patient samples [76], and GSE53478 for NK-cell line YT. These gene expression microarray profiling data were generated using the HG U133 Plus 2.0 gene chip (Affymetrix). Expression values were given as boxplots using R-packages (http://www.bioconductor.org/). Statistical significance was calculated by the Mann-Whitney U Test. P-values less than 0.05 were indicated by an asterisk.

For additional analyses of differential gene activities the data were transformed as follows: After RMA-background correction and quantile normalization of the spot intensities, the profiling data were expressed as ratios of the sample mean and subsequently log2 transformed. Data processing was performed via R/Bioconductor using limma and affy packages. To parse biological function of shortlisted genes, gene-annotation enrichment analysis was performed using DAVID bioinformatics resources [77].

To search potential binding sites of micro-RNAs in the 3’-UTRs of particular gene candidates we used the online tool TargetScan (www.targetscan.org/vert_71/).

### Polymerase chain-reaction (PCR) analyses

Total RNA was extracted from cell line samples using TRIzol reagent (Invitrogen, Darmstadt, Germany). Primary human total RNA used in this study was commercially obtained - isolated from NK-cells (3H Biomedical, Uppsala, Sweden), and RNA from CD3-positive T-cells, CD19-positive B-cells and CD34-positive HSCs from Miltenyi Biotec (Bergisch Gladbach, Germany). The primary cells were not treated with interleukins before harvesting their RNA. cDNA was synthesized from 5 μg RNA by random priming using Superscript II (Invitrogen). Real-time quantitative (RQ)-PCR analysis was performed with the 7500 Real-time System, using commercial buffer and primer sets (Applied Biosystems/Life Technologies, Darmstadt, Germany). Quantification of MSX1 was performed as described previously [20]. For normalization of expression levels we analyzed the transcript of TATA box binding protein (TBP).

For copy number quantification genomic DNA was extracted using Qiagen Gentra Puregene Kit (Qiagen). Oligonucleotides used for analysis of MSX1 and MEF2C (normalization) were as described previously [20], those for AUTS2 were as follows: forward exon1 5’-GAACCATGGATGGCCCGACG-3’, reverse exon1 5’-GACGACGAGGCGAGTGAGAG-3’, forward exon3 5’-TCAAGCCAGGACAGAACAGCTG-3’, reverse exon3 5’-ATCACTGAGCCTTTCCCGAGAG-3’, those for IRF4 were: forward 5’-GGCGGAGAGTTCGGCATGAG-3’, reverse 5’-GATGCGGAAGATGCTCTTCTCC-3’, and those for PRDM1 were: forward 5’-GATGCGGATATGA-CTCTGTGGAC-3’, reverse 5’-AAGGATGCCTCCGCCT-GAACCG-3’. The oligonucleotides were obtained from Eurofins MWG (Ebersbach, Germany). Quantitative analyses were performed in triplicate. Standard deviations are presented in the figures as error bars. The statistical significance was assessed by ANOVA and the calculated p-values indicated by asterisks (* p<0.05, ** p<0.01, *** p<0.001, n.s. not significant).

Qualitative reverse transcription (RT)-PCR was performed using taqpol (Qiagen) and the thermocycler TGradient (Biometra, Göttingen, Germany). The oligonucleotides used for analysis of AUTS2 and YY1 (control) were as follows: AUTS2-forward 5’- AGTCCACCTCGGCAGAAGAGG-3’, AUTS2-reverse 5’-CTTCCTGGTCACTGTCACTGTC-3’, YY1- forward 5’- AAGCAGGTGCAGATCAAGAC-3’, YY1-reverse 5’- CCGAGTTATCCCTGAACATC-3’. The oligonucleotides were obtained from Eurofins MWG. The generated PCR products were subsequently analyzed by agarose gel electrophoresis. Documentation was performed using the Azure c200 Gel Imaging System (Azure Biosystems, Dublin, CA, USA). Cloning of the PCR products was performed using vector pCR4-TOPO according to the manufacturer’s protocol (ThermoFisher, Dreieich, Germany), and sequencing of the inserts at Eurofins MWG. For each PCR product we sequenced at least 4 clones in both directions. Sequence analysis was performed using BLAST (NIH/NCBI).

### Protein analyses

Western blots were generated by the semi-dry method. Protein lysates from cell lines were prepared using SIGMAFast protease inhibitor cocktail (Sigma). Proteins were transferred onto nitrocellulose membranes (Bio-Rad, München, Germany) and blocked with 5% dry milk powder dissolved in phosphate-buffered-saline buffer (PBS). The following antibodies were used: alpha-Tubulin (Sigma) and IRF4 (NOVUS Biologicals, Colorado, USA). For loading control blots were reversibly stained with Poinceau (Sigma) and detection of alpha-Tubulin (TUBA) was performed thereafter. Secondary antibodies were linked to peroxidase for detection by Western-Lightning-ECL (Perkin Elmer, Waltham, MA, USA). Documentation was performed using the digital system ChemoStar Imager (INTAS, Göttingen, Germany).

### Reporter gene assay

For creation of reporter gene constructs we combined a reporter with a regulatory genomic fragment derived from the first intronic region of MSX1, containing a potential binding site for IRF4 [41]. We cloned the genomic PCR product of the corresponding intron region (regulator) and of the HOXA9 gene, comprising exon1-intron1-exon2 (reporter), into the *Hind*III/*Bam*HI and *Eco*RI sites, respectively, of the expression vector pcDNA3 downstream of the CMV enhancer. The oligonucleotides used for the amplification of the MSX1-regulator were obtained from Eurofins MWG. Their sequences were as follows: MSX1-for 5’-ATAAGCTTCACCCCAGCGGATGAATGTGTG-3’, MSX1-rev 5’-TAGGATCCTGTAGGAGCTTCGGAACCTC-3’. Introduced restriction sites used for cloning are underlined. Constructs were validated by sequence analysis (Eurofins MWG). Transfections of plasmid-DNA into NIH-3T3 cells (DSMZ ACC 59) were performed using SuperFect Transfection Reagent (Qiagen). Commercial HOXA9 and TBP assays were used for RQ-PCR to quantify the spliced reporter-transcript, corresponding to the regulator activity. A cotransfected commercial luciferase construct served as transfection control and was quantified by the Luciferase Assay System (Promega, Mannheim, Germany) using the luminometer Lumat LB9501 (Berthold Technologies, Bad Wildbad, Germany).

### MTT assay

Cell lines were treated for 2 days with 10 μg/ml TSA (Sigma) and 0.5 μM ICBP112 (Sigma) which have been dissolved in dimethylsulfoxide, and subsequently prepared for standardized MTT (3-(4,5-dimethylthiazol-2-yl)-2,5-diphenyltetrazolium bromide; obtained from Sigma) assays. The measurement was performed twice in triplicates. The absorbance was determined at 540 nm and at 620 nm as background control using ELISA reader Multiskan EX (Thermo Electron, Vantaa, Finland).

## SUPPLEMENTARY MATERIALS FIGURES AND TABLE


