# Supplementary material for: NKL homeobox gene MSX1 acts like a tumor suppressor in NK-cell leukemia
Source: Oncotarget. 2017 Jun 21;8(40):66815–32. doi: 10.18632/oncotarget.18609 (PMC5620138; doi:10.18632/oncotarget.18609)
Supplement: Supplementary file 1 [file oncotarget-08-66815-s001.pdf]

NKL homeobox gene MSX1 acts like a tumor suppressor in NK-cell leukemia

SUPPLEMENTARY MATERIALS

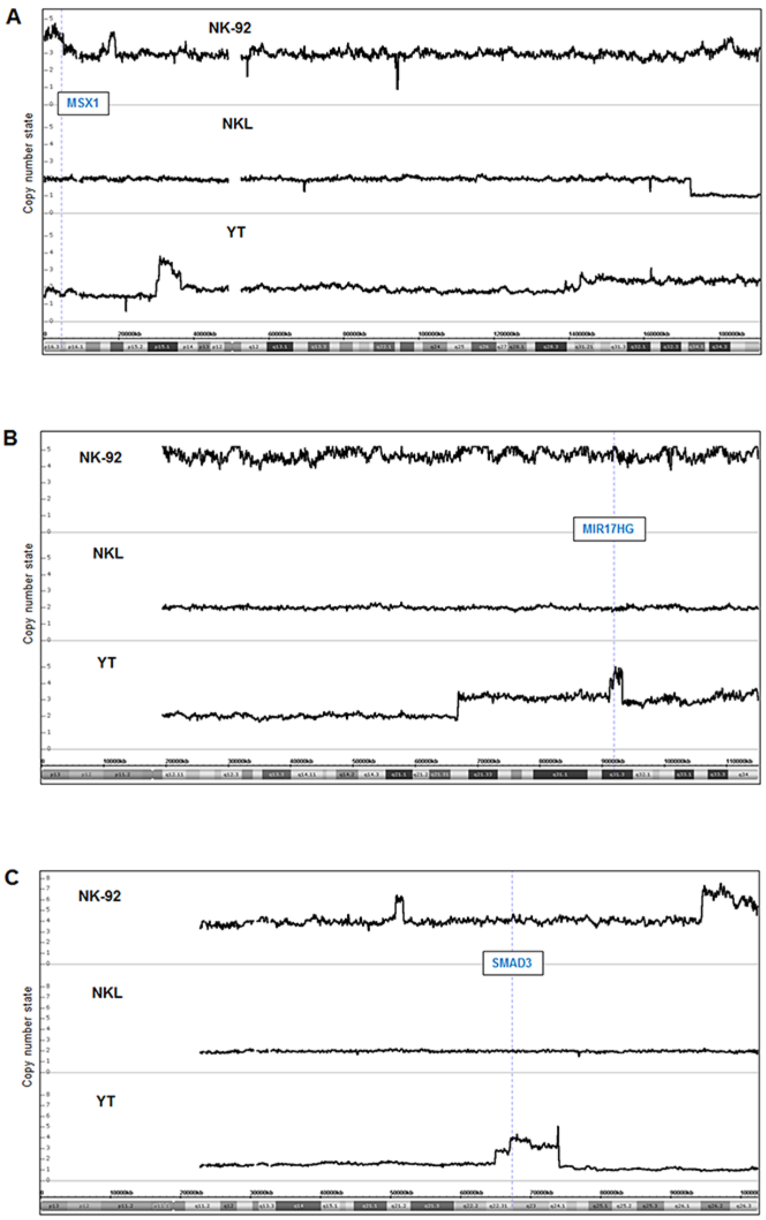

Supplementary Figure 1: Genomic profiling data. Data from selected chromosomes are shown to indicate copy number alterations and particular gene loci, including **(A)** MSX1, **(B)** MIR17HG, **(C)** SMAD3. (*Continued*)

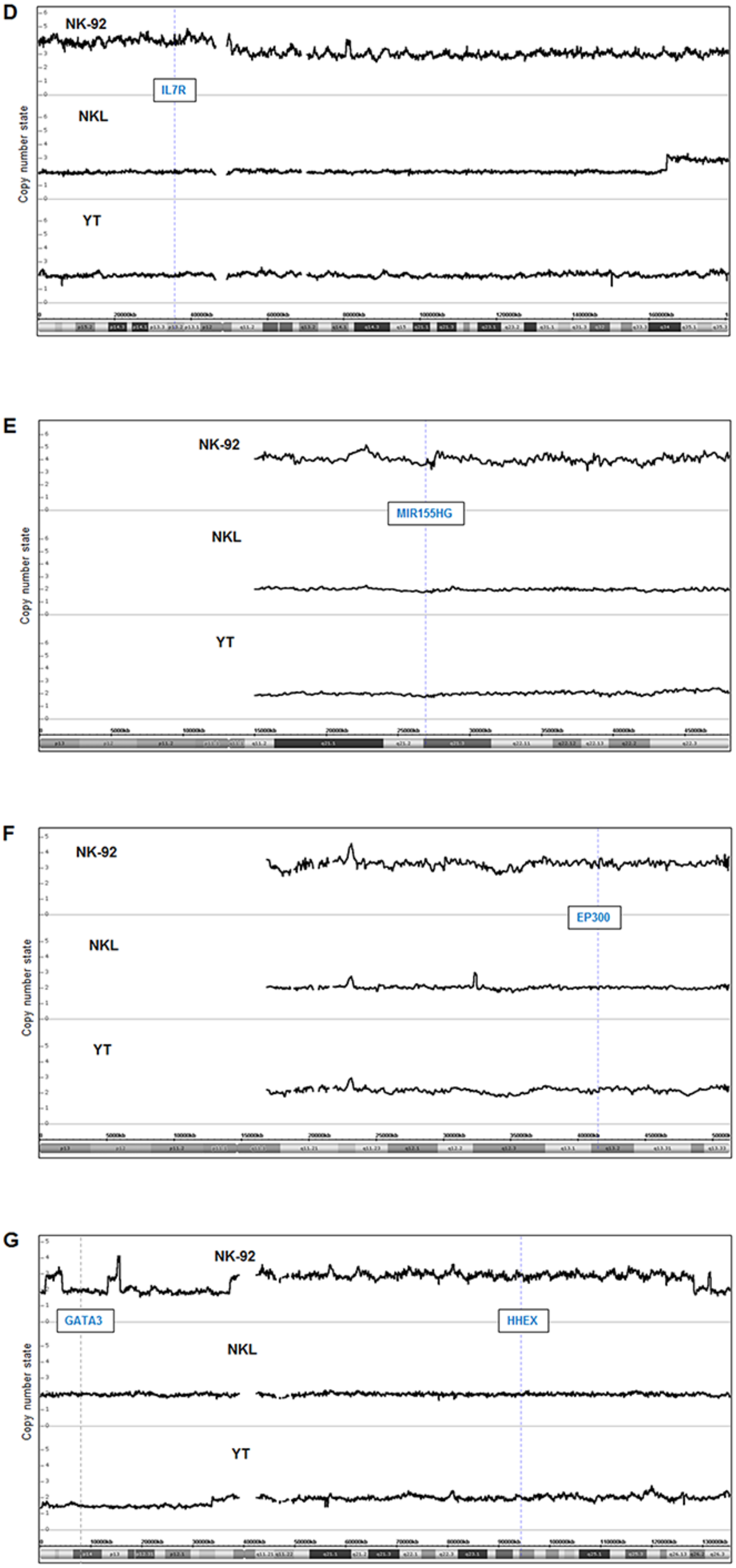

Supplementary Figure 1: Genomic profiling data. (***Continued***)Data from selected chromosomes are shown to indicate copy number alterations and particular gene loci, including **(D)** IL7R, **(E)** MIR155HG, **(F)** EP300, **(G)** GATA3 and HHEX.

Category	Term	RT	Genes	Count	%	P-Value	Benjamini
KEGG_PATHWAY	Metabolic pathways	RT		79	9,7	2,5E-2	2,8E-1
KEGG_PATHWAY	Cell cycle	RT		36	4,4	2,1E-17	5,6E-15
KEGG_PATHWAY	Biosynthesis of antibiotics	RT		24	2,9	5,6E-4	2,1E-2
KEGG_PATHWAY	HTLV-I infection	RT		24	2,9	6,5E-3	1,2E-1
KEGG_PATHWAY	Epstein-Barr virus infection	RT		21	2,6	1,8E-3	5,2E-2
KEGG_PATHWAY	Viral carcinogenesis	RT		21	2,6	4,4E-3	9,2E-2
KEGG_PATHWAY	Oocyte meiosis	RT		20	2,4	2,4E-6	3,2E-4
KEGG_PATHWAY	Purine metabolism	RT		20	2,4	1,8E-3	5,6E-2
KEGG_PATHWAY	Huntington's disease	RT		18	2,2	2,1E-2	2,5E-1
KEGG_PATHWAY	RNA transport	RT		17	2,1	1,6E-2	2,2E-1
KEGG_PATHWAY	Ribosome	RT		16	2,0	4,2E-3	9,6E-2
KEGG_PATHWAY	Biosynthesis of amino acids	RT		15	1,8	2,0E-5	1,8E-3
KEGG_PATHWAY	Carbon metabolism	RT		15	1,8	1,9E-3	4,9E-2
KEGG_PATHWAY	p53 signaling pathway	RT		14	1,7	3,0E-5	2,0E-3
KEGG_PATHWAY	Progesterone-mediated oocyte maturation	RT		14	1,7	4,7E-4	2,0E-2
KEGG_PATHWAY	Protein processing in endoplasmic reticulum	RT		14	1,7	9,7E-2	6,0E-1
KEGG_PATHWAY	Pyrimidine metabolism	RT		13	1,6	7,1E-3	1,2E-1
KEGG_PATHWAY	Hepatitis B	RT		13	1,6	7,1E-2	5,7E-1
KEGG_PATHWAY	Oxidative phosphorylation	RT		12	1,5	8,2E-2	5,9E-1
KEGG_PATHWAY	Sphingolipid signaling pathway	RT		11	1,3	9,1E-2	6,2E-1

Category	Term	RT	Genes	Count	%	P-Value	Benjamini
KEGG_PATHWAY	Pathways in cancer	RT		23	3,5	1,5E-2	1,2E-1
KEGG_PATHWAY	Natural killer cell mediated cytotoxicity	RT		20	3,0	2,5E-8	5,5E-6
KEGG_PATHWAY	Chemokine signaling pathway	RT		18	2,7	2,0E-4	8,7E-3
KEGG_PATHWAY	Osteoclast differentiation	RT		17	2,6	9,1E-6	9,7E-4
KEGG_PATHWAY	FoxO signaling pathway	RT		16	2,4	4,9E-5	3,5E-3
KEGG_PATHWAY	MAPK signaling pathway	RT		16	2,4	2,9E-2	1,7E-1
KEGG_PATHWAY	Tuberculosis	RT		15	2,3	2,9E-3	4,4E-2
KEGG_PATHWAY	Endocytosis	RT		15	2,3	5,9E-2	2,6E-1
KEGG_PATHWAY	Leukocyte transendothelial migration	RT		14	2,1	1,9E-4	9,9E-3
KEGG_PATHWAY	Rap1 signaling pathway	RT		14	2,1	2,8E-2	1,8E-1
KEGG_PATHWAY	Non-alcoholic fatty liver disease (NAFLD)	RT		13	2,0	5,5E-3	6,8E-2
KEGG_PATHWAY	Focal adhesion	RT		13	2,0	5,0E-2	2,5E-1
KEGG_PATHWAY	Regulation of actin cytoskeleton	RT		13	2,0	5,8E-2	2,6E-1
KEGG_PATHWAY	Ras signaling pathway	RT		13	2,0	8,6E-2	3,1E-1
KEGG_PATHWAY	Cytokine-cytokine receptor interaction	RT		13	2,0	9,5E-2	3,2E-1
KEGG_PATHWAY	T cell receptor signaling pathway	RT		12	1,8	7,5E-4	1,8E-2
KEGG_PATHWAY	Sphingolipid signaling pathway	RT		12	1,8	2,6E-3	4,6E-2
KEGG_PATHWAY	Proteoglycans in cancer	RT		12	1,8	8,1E-2	3,0E-1
KEGG_PATHWAY	NF-kappa B signaling pathway	RT		11	1,7	7,3E-4	2,0E-2
KEGG_PATHWAY	Neurotrophin signaling pathway	RT		11	1,7	7,9E-3	8,2E-2
KEGG_PATHWAY	Adrenergic signaling in cardiomyocytes	RT		11	1,7	2,8E-2	1,8E-1
KEGG_PATHWAY	Transcriptional misregulation in cancer	RT		11	1,7	6,3E-2	2,6E-1
KEGG_PATHWAY	Influenza A	RT		11	1,7	7,6E-2	2,9E-1
KEGG_PATHWAY	Fc epsilon RI signaling pathway	RT		10	1,5	4,8E-4	1,7E-2
KEGG_PATHWAY	B cell receptor signaling pathway	RT		10	1,5	5,3E-4	1,6E-2
KEGG_PATHWAY	Fc gamma R-mediated phagocytosis	RT		10	1,5	2,2E-3	4,2E-2
KEGG_PATHWAY	Choline metabolism in cancer	RT		10	1,5	7,6E-3	8,7E-2
KEGG_PATHWAY	Chagas disease (American trypanosomiasis)	RT		10	1,5	9,1E-3	9,0E-2
KEGG_PATHWAY	Platelet activation	RT		10	1,5	3,4E-2	1,9E-1
KEGG_PATHWAY	Tight junction	RT		10	1,5	4,5E-2	2,3E-1
KEGG_PATHWAY	Cell adhesion molecules (CAMs)	RT		10	1,5	5,5E-2	2,6E-1
KEGG_PATHWAY	Hepatitis B	RT		10	1,5	6,1E-2	2,5E-1
KEGG_PATHWAY	Oxytocin signaling pathway	RT		10	1,5	9,3E-2	3,2E-1

Supplementary Figure 2: Gene-annotation enrichment analysis data (DAVID). The top 1000 differentially expressed genes between NK-cell lines and primary NK-cells were analyzed to identify deregulated cell functions according to KEGG pathways. Aberrantly activated functions are shown above, suppressed functions below.

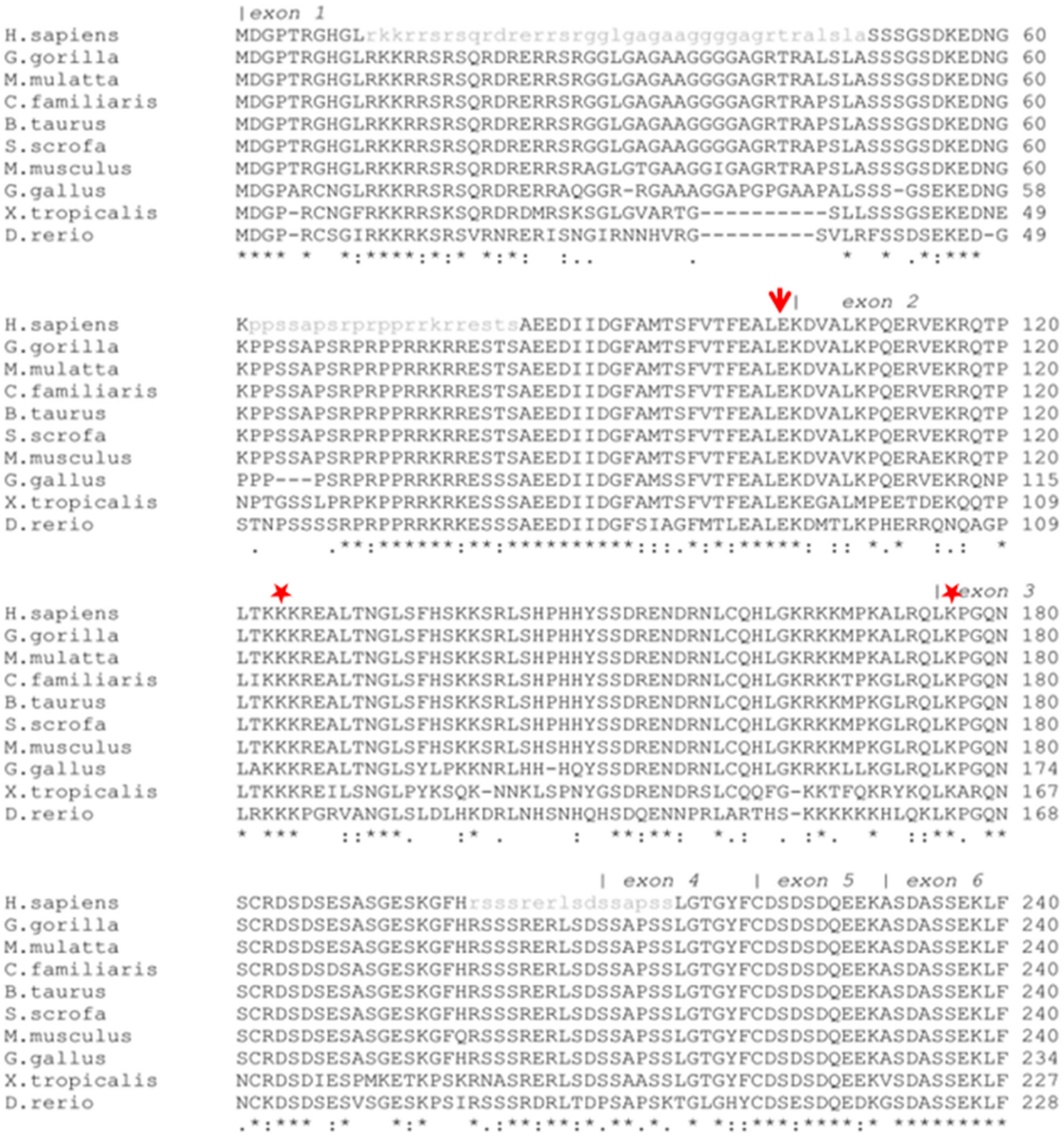

Supplementary Figure 3: AUTS2 alignment. The illustrated alignment was obtained from Beunders et al. (2013) [50], showing the N-terminal part of AUTS2 from the indicated vertebrates. Conserved amino acid residues are indicated by an asterisk, similar residues by dots. The red stars indicate the here identified mutated positions, the red arrow marks the position of deduced protein truncation.

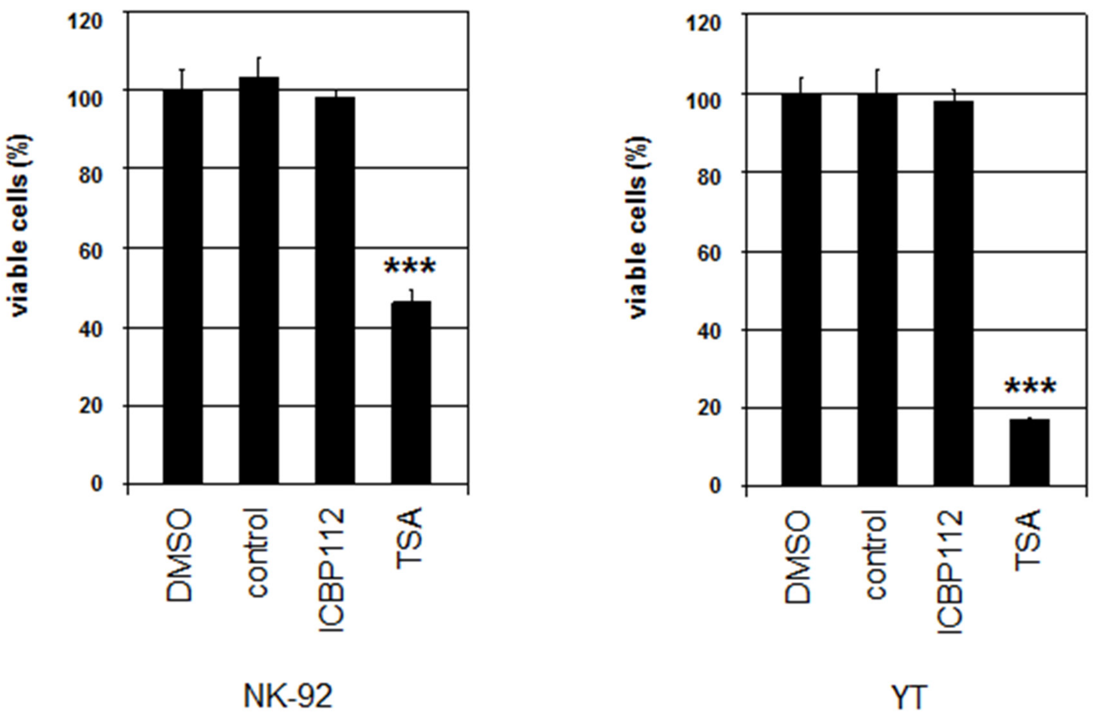

Supplementary Figure 4: Cell viability analysis. MTT assay was performed in NK-92 (left) and YT (right) after treatment with histone acetyltransferase inhibitor ICBP112 and histone deacetylase inhibitor TSA. The statistical significance is indicated by asterisks.

Supplementary Table 1: Comparative expression profiling. The data demonstrate gene activities from primary NK-cells and NK-cell lines, showing (A) overexpressed and (B) downregulated genes between these two groups. Genes analyzed in this study are indicated in red and green, respectively.

See Supplementary Files 1A and 1B
